# Editorial: Delayed Injury Mechanisms After Ischemic and Hemorrhagic Stroke

**DOI:** 10.3389/fneur.2022.881629

**Published:** 2022-03-18

**Authors:** Devin W. McBride, Prativa Sherchan, Qin Hu, Budbazar Enkhjargal

**Affiliations:** ^1^The Vivian L. Smith Department of Neurosurgery, The University of Texas Health Science Center at Houston, Houston, TX, United States; ^2^Department of Basic Sciences, Loma Linda University, Loma Linda, CA, United States; ^3^School of Medicine, Shanghai Jiao Tong University, Shanghai, China; ^4^School of Medicine, Whitaker Cardiovascular Institute, Boston University, Boston, MA, United States

**Keywords:** delayed injury, stroke, hemorrhage–cerebral, cerebral ischemia, biomarkers

Stroke is a leading cause of death worldwide with about 12 million new cases per year. To date, no one is able to predict when a stroke will occur. Although “time is brain” has been the slogan for stroke care, many patients still arrive for care outside of the desired time window. Thus, research endeavors have focused on developing treatments to restore injured brain tissue. With the exception of a few treatments, many drug candidates fail in clinical trials. To better understand the pathological events following stroke, as well as to identify novel therapeutic targets, research focused on delayed injury mechanisms is ongoing.

Inflammation, edema, and vascular dysfunction are crucial pathophysiological events involved in delayed injury after stroke. While considerable research has been undertaken to understand these damaging processes in the acute phase, less is known about their respective contributions to delayed injury occurring 4–14 days after stroke. Furthermore, different stroke subtypes (i.e., ischemic vs. hemorrhagic) have similar, yet slightly differing delayed injury mechanisms.

The goal of this Research Topic is to provide a collection of papers investigating delayed injury mechanisms after stroke. Numerous experimental papers focus on the acute injury phase following stroke, usually <3 days. However, research looking at the role of these pathological events causing and during the delayed phase (typically 3–10 days in animals, 5–14 days in humans) is limited, especially with respect to experimental studies. This collection will promote the importance of delayed injury as an injury phase of stroke which can be prevented, thereby promoting recovery from the initial stroke (ischemic or hemorrhagic) injury. Naturally, research regarding delayed injury continues to examine potential biomarkers and predictors which can be used to identify patients at-risk for delayed injury.

## Delayed Injury in Ischemic Stroke

Shen et al. investigate the utility of various hyperglycemia ratios in predicting poor outcome after ischemic stroke. The prospective study observed that using the ratio of fasting glucose to glycated hemoglobin (assessed by 48 h post-injury) is independently associated with worse outcomes at 3 and 6 months.

## Delayed Injury in Intracerebral Hemorrhage

In patients with intracerebral hemorrhage, peri-hematoma cerebral blood flow assessed 6 h post-hemorrhage was reported to be an independent risk factor for hematoma expansion in a delayed fashion. The results of the study by Wang et al. suggest closely monitoring cerebral blood flow in the peri-hematoma tissue. At a more delayed time point, Haque et al. performed a longitudinal imaging study to determine the volumes of the peri-hematoma tissue and hematoma, and microstructural integrity. The findings suggest that imaging at 1 month post-intracerebral hemorrhage can predict patients that are at-risk for poor outcomes at 3 and 6 months.

## Delayed Injury in Subarachnoid Hemorrhage

Delayed injury following subarachnoid hemorrhage manifests as delayed cerebral ischemia and neurological decline. To date there exists many studies which have investigated various factors for their use as predictors of delayed cerebral ischemia. While not an exhaustive list, hematoma volume, inflammation, vasospasm, and other serum cytokines/chemokines/factors have been reported. Some of the factors reported can easily be obtained (e.g., clinical data/characteristics, routine labs), while others (e.g., cytokines, serum proteins) require special methods. The study by Csók et al. in this topic develops a risk prediction model using easily obtained/available clinical data. The authors observed that their model, which combines hematoma volume, level of consciousness, and sonographic mean flow velocity of the intracranial arteries from admission to post-bleed day 5, is a simple and precise method for identifying subarachnoid hemorrhage patients which are at-risk for delayed cerebral ischemia. Another study in this topic also investigated readily available clinical data for predicting delayed cerebral ischemia. In their retrospective study, Lin et al. examined clinical characteristics and laboratory data to identify factors which can predict development of cerebral infarction in elderly subarachnoid hemorrhage patients. Of the factors assessed, admission body temperature was found to be independently associated with cerebral infarction. The findings from the study suggest that patients who have an admission body temperature lower than 36.6°C are at increased risk for developing cerebral infarction than patients who have an admission body temperature <36.6°C. The final manuscript investigating biomarkers/factors which can predict development of delayed cerebral ischemia examines inflammatory data. In a prospective study, Gusdon et al. observed that male subarachnoid hemorrhage patients had a higher number of monocytes than females. Furthermore, in males, early elevation of monocytes can predict delayed cerebral ischemia and poor outcomes.

Microthrombi and microvessel dysfunction have been receiving more attention as evidence of their contributions to delayed cerebral ischemia increase. In this topic, Pang et al. perform a mechanistic experimental study investigating microthrombi and pericytes. In agreement with others, microthrombi and microvessel dysfunction are factors contributing to poor outcome after subarachnoid hemorrhage. The work presented by Pang et al. shows that microvessels containing microthrombi have reduced pericyte coverage which may contribute to vasculature dysfunction. This study suggests that there may be a direct connection between microthrombi and microvessel dysfunction *via* pericytes and P-selectin. Additionally, the authors provide evidence that ApoE deficiency may contribute to more extensive damage *via* pericyte loss and microthrombi formation. Future studies need to be performed to investigate if reduced pericyte coverage has any effect on delayed injury.

In the future, understanding precise molecular and pathophysiological mechanism of delayed brain injury after stroke is crucial as a key of diagnostic and therapeutic strategy. Perspective studies require to fill this gap in stroke study as well as to discover successful drug candidates for the patients.

## Author Contributions

All authors listed have made a substantial, direct, and intellectual contribution to the work and approved it for publication.

## Funding

DWM was funded by grants from the NIH and the Brain Aneurysm Foundation.

## Conflict of Interest

The authors declare that the research was conducted in the absence of any commercial or financial relationships that could be construed as a potential conflict of interest.

## Publisher's Note

All claims expressed in this article are solely those of the authors and do not necessarily represent those of their affiliated organizations, or those of the publisher, the editors and the reviewers. Any product that may be evaluated in this article, or claim that may be made by its manufacturer, is not guaranteed or endorsed by the publisher.

